# Enhanced function of vaccine dendritic cells from obese donors upon inhibition of the lipid metabolism

**DOI:** 10.1002/ctm2.557

**Published:** 2022-02-25

**Authors:** Chiara Massa, Barbara Seliger

**Affiliations:** ^1^ Institute of Medical Immunology Martin Luther University Halle‐Wittenberg Halle (Saale) Germany; ^2^ Fraunhofer Institute for Cell therapy and Immunology Leipzig Germany

Dear Editor,

This study demonstrates that the inhibition of the lipid metabolism during in vitro differentiation of monocytes toward dendritic cells (DC) enhances their functional interaction with immune effector cells, thus providing a way to improve the clinical implementation of vaccine DC for example in tumour immunotherapy.^1^ This enhanced functionality was stronger using monocytes from obese donors, a population known for its low response to vaccination^2^ as well as higher risk of cancer.^3^

In recent years there has been a focus on immunometabolism^4^ as a way to improve immune cell functions in the setting of (tumour) immunotherapy. Due to the renewed interest in the active vaccination of cancer patients for example in combination with checkpoint inhibitors, metabolic ways to improve the functionality of DC‐based vaccines were investigated. To this purpose monocytes from healthy donors were differentiated in vitro using the 2‐day long protocol of FastDC^5^ and then stimulated with the clinical gold standard or the MPLA cocktail as previously described.^6^ Mature FastDC were then evaluated for phenotype, metabolic properties and functional interaction with immune cells.

Regarding their metabolism, maturations of FastDC did not influence the uptake of glucose, whereas it enhanced fatty acid (FA) uptake, particularly in the highly functional MPLA FastDC both during (Figure 1A) and at the end of maturation (Figure 1B) leading to an enhanced lipid content (Figure 1C). MPLA FastDC had also highly active mitochondria as highlighted by the enhanced oxygen consumption (Figure 1D), but low levels of oxygen reactive species (Figure 1E) suggesting the activation of detoxifying systems.

**FIGURE 1:**
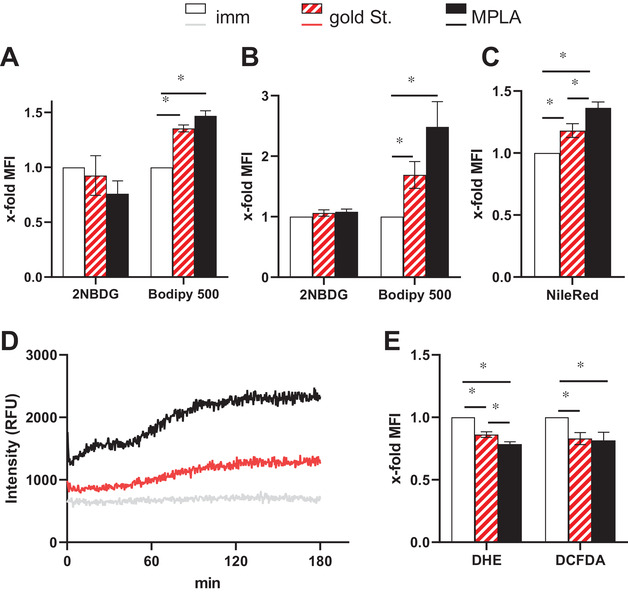
Metabolic characteristics of FastDC. (A) Differentiated FastDC were incubated with the clinical gold standard or the MPLA maturation cocktails as well as with the glucose analogue 2‐NBDG or the fluorescent fatty acid analogue Bodipy500. After 2 h metabolite uptake was evaluated by flow cytometry. Shown are the mean ± SE of the x‐fold increase in MFI over immature FastDC from three different experiments. (B‐E) FastDC at the end of the maturation were evaluated for their metabolic properties. FastDC were incubated for 10 min at 37°C with 2‐NBDG and Bodipy500 to evaluate metabolite uptake (B) as well as with NileRed to determine the content in neutral lipid (C). Mitochondria oxygen consumption was evaluated using the MitoXpress Xtra assay from Agilent following manufacturer's instruction and using 4 × 10^5^ FastDC/well in PBS (D). FastDC were incubated for 10 min at 37°C with 2‐dihydro ethidiumbromide (DHE) (Cayman Chemical) or dichlor‐dihydrofluorescein‐diacetate (DCFDA) (Sigma Aldrich) to evaluate the presence of oxygen reactive species (E). Shown are the mean ± SE of the x‐fold increase in MFI over immature FastDC from 8 different donors (B, C and E) and one out of five experiments with similar results (D). A‐C and E: *, *p* < 0.05 using the paired ANOVA test

To evaluate the role of the 'lipid phenotype' for MPLA FastDC functionality, the anti‐obesity drug orlistat was implemented. Its presence during MPLA FastDC maturation resulted in a slightly lower upregulation of costimulatory and adhesion molecules (Figure S1A), but did not affect their interaction with immune effector cells evaluated as degranulation to different targets or as secretion of IFN‐γ (Figure S1B‐D). Stronger effects were observed when orlistat was added from the start of the in vitro culture, which were not due to impaired differentiation, but resulted in a significantly reduced expression of all maturation markers (Figure 2A). Unexpectedly, orlistat‐treated MPLA FastDC had a stronger capability to induce immune effector cells to degranulate (Figure 2B,C), secrete IFN‐γ (Figure 2D) and proliferate (Figure 2E). These enhanced functional capabilities were not present when orlistat was combined with the gold standard maturation cocktail (Figure S2) indicating that some properties provided by the MPLA cocktail were required. Evaluation of the secretory properties of MPLA FastDC upon orlistat treatment highlighted a significant loss of both IL‐12p70 and IL‐10 (Figure S3A,B) with a non‐significant trend of reduced IL‐12p70/IL10‐ratio in the overall cohort (Figure S3C). However, when the donors were divided based on their body mass index (BMI), all obese donors responded to orlistat with a significant increase in the IL‐12p70/IL‐10 ratio as well as an enhanced IFN‐γ secretion by CD56^br^ NK cells (Figure 2F,G).

**FIGURE 2:**
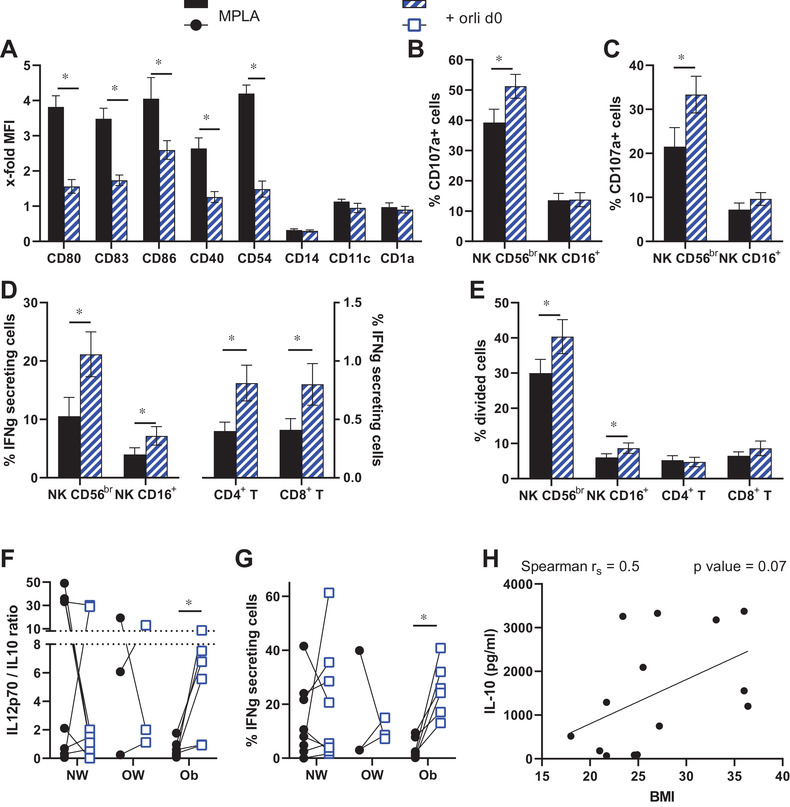
Effects of orlistat presence during differentiation on MPLA FastDC phenotype and function. MPLA FastDC were differentiated in the absence or presence of 50 μM orlistat (+ orli d0). (A) Expression of the indicated molecules on mature FastDC was determined by flow cytometry and is shown as the mean ± SE of the x‐fold increases in MFI over immature FastDC from 5 to 17 different donors. (B‐D) Autologous PBL were co‐cultured with the different MPLA FastDC for 18 h and evaluated for their ability to degranulate in response to a 4 h co‐culture with K562 (B) or the renal cell carcinoma cell line RCC53 (C) as well as to secrete IFN‐γ (D). Shown are the mean ± SE of the percentages of degranulating / IFN‐γ secreting cells among the indicated immune effector cells from 17 different donors. Evaluation of secretion in CD4^+^ and CD8^+^ T cells was performed in nine donors. (E) PBL were stained with the proliferation dye‐647 and cultured with the different MPLA FastDC for 5 days. Shown are the percentages of divided cells among the different immune populations from nine different donors. (F‐G) The IL‐12p70/IL‐10 ratio and the percentages of IFN‐γ secreting CD56^br^ NK cells of MPLA FastDC differentiated or not with orlistat are shown for each individual donor (*n* = 17) upon subdivision based on their BMI into normal‐ (NW), over‐weight (OW) or obese (Ob). (H) The correlation between the IL‐10 secreted by FastDC in response to MPLA stimulation, and the donor BMI is shown together with its spearman coefficient and *p* value. A‐G: *, *p* < 0.05 in paired t‐test for orlistat treatment

Due to the positive even if not statistically significant association between the donor BMI and the IL‐10 secreted by MPLA FastDC (Figure 2H), the effects of a blocking antibody (Ab) against IL‐10 were compared to those of orlistat implementing exclusively FastDC from obese donors. Addition of the blocking Ab during the co‐culture of MPLA FastDC with the effector cells did not affect their degranulation (Figure 3A,B), but enhanced the IFN‐γ secretion of CD56^br^ NK cells, without reaching the levels of orlistat (Figure 3C). Addition of the Ab during the differentiation or maturation of FastDC did not alter the expression of costimulatory molecules, but significantly enhanced IL‐12p70 secretion (Figure 3D,E). Functionally, these FastDC were similar to orlistat for the effects on NK cell degranulation, but not regarding their secretion of IFN‐γ (Figure 3F‐H). Comparison of five donors, in which the Ab was investigated at all three different time points, demonstrated no significant differences (Figure S4) indicating that the high levels of IL‐10 secreted by obese donors' FastDC have both autocrine and paracrine inhibitory effects, as confirmed by supplementation of recombinant IL‐10 to orlistat treatment (Figure S5). However, the IL‐10 reduction is not the solely mechanism, through which orlistat enhances FastDC immunogenicity.

**FIGURE 3:**
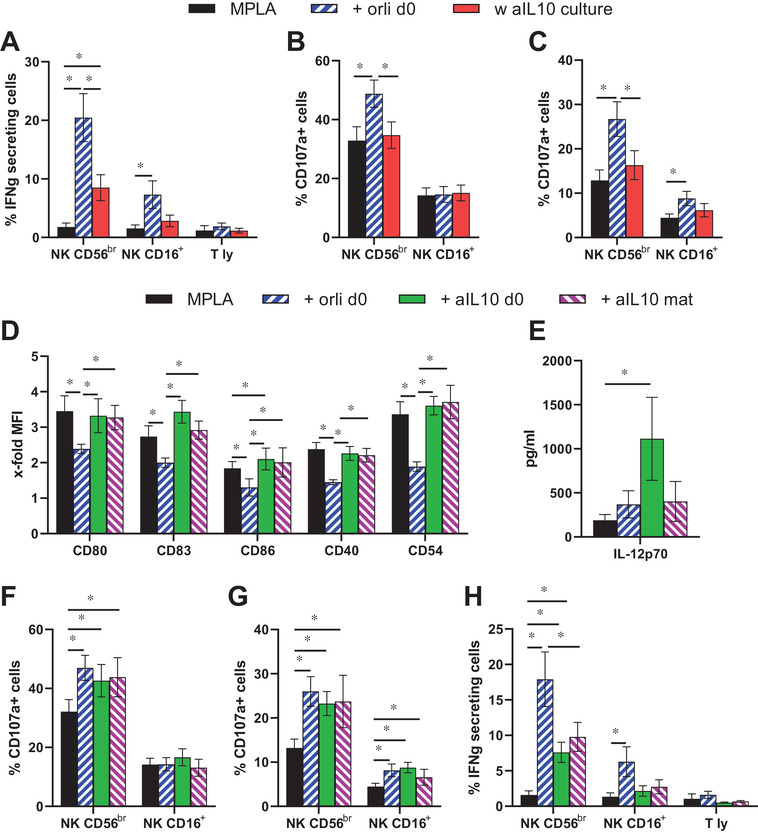
Role of IL‐10 in the functionality of obese MPLA FastDC. (A‐C) FastDC from obese donors were matured with the MPLA cocktail and incubated with autologous PBL in the presence or absence of 10 μg/ml blocking anti‐IL‐10 Ab. MPLA FastDC differentiated in the presence of orlistat were also implemented as comparison. Shown are the percentages of immune cells secreting IFN‐γ (A) and degranulating in response to K562 (B) or RCC53 (C) from 11 different donors. (D‐H) The blocking anti‐IL‐10 Ab was added to obese FastDC either from the start of differentiation (+ aIL‐10 d0, eight different donors) or together with the maturation cocktail (+ aIL‐10 mat, seven different donors). FastDC differentiated in the presence of orlistat and matured with the MPLA cocktail were implemented as comparison. (D) Expression of maturation markers are shown as x‐fold increase in MFI over immature FastDC. (E) Concentration of IL‐12p70 in FastDC supernatants from all the donors as determined by ELISA. (F‐H) PBL were incubated for 18 h with autologous FastDC treated with the blocking anti‐IL‐10 Ab or orlistat. Percentages of the different immune subsets secreting IFN‐γ (F) or degranulating in response to K562 (G) or RCC53 cells (H) are shown as mean ± SE from all the evaluated donors (i.e., *n* = 8 for 'aIL‐10 d0' and *n* = 7 for 'aIL‐10 mat'). A‐H: *, *p* < 0.05 in ANOVA paired test

Since orlistat affects FA metabolism inhibiting both the FA synthase (FASN) and different lipases,^7^ C75 and Cay10499 were implemented as additional inhibitors of FASN (with also effects on carnitine O‐palmitoyltransferase‐1^8^) and multiple lipases, respectively. The three inhibitors similarly reduced FastDC expression of costimulatory molecules (Figure 4A) and IL‐10 secretion (Figure 4B). Regarding IL‐12p70 secretion, C75 strongly reduced it in all donors, whereas orlistat and Cay10499 exhibited both positive and negative effects, but with various donors responding in opposite ways to the two inhibitors (Figure 4C). Independently of the effect on IL‐12p70, its ratio to IL‐10 was enhanced by all inhibitors, but significantly only for orlistat and Cay10499 (Figure 4D). Functionally, C75 and Cay10499 treatment (Figure 4E) or their combination (data not shown) enhanced IFN‐γ secretion by CD56^br^ NK cells, but not to the levels of orlistat. Interestingly, Cay10499 induced higher percentages of CD4^+^ and CD8^+^ T cells to secrete IFN‐γ than orlistat (Figure 4E), but no significant effects were found on their proliferation (data not shown). Evaluation of NK cell cytolytic activity demonstrated negligible effects of C75, whereas Cay10499 enhanced their degranulation similarly to orlistat (Figure 4F,G).

**FIGURE 4:**
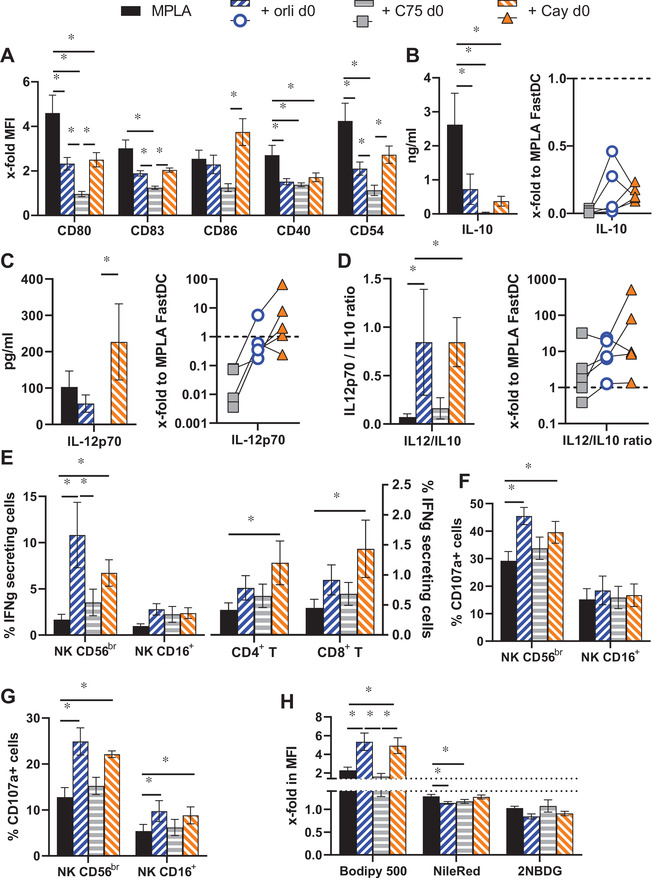
Comparison of the role of FASN and lipases in the activity of orlistat. Obese monocytes were differentiated in the presence of Orlistat, C75 (50 μM) or Cay10499 (10 μM) before maturation with the MPLA cocktail. (A‐D) Mature FastDC were evaluated for expression of costimulatory molecules by flow cytometry (A) and their supernatants characterised by ELISA for IL‐10 (B) and IL‐12p70 content (C), from which the IL‐12p70 to IL‐10 ratio was calculated (D). (E‐G) PBL were co‐cultured with the different MPLA FastDC and evaluated after 18 h for the percentages of cells that secrete IFN‐γ (E) or degranulate in response to K562 (F) or RCC53 cells (G). (H) FastDC were also evaluated for the uptake of Bodipy500 and 2‐NBDG as well as stained with NileRed. Dotted lines highlight the brake of the y‐axis. Shown are the mean ± SE from five different donors. For the ELISA data, cytokine levels upon inhibitor treatment were normalised to untreated MPLA and are shown for each individual donor as x‐fold over MPLA FastDC (set to 1, dashed line). A‐H: *, *p* < 0.05 in ANOVA test

Characterization of the metabolic consequences of the different inhibitors highlighted no influence on glucose internalisation (Figure 4H), whereas both 'lipase' inhibitors further enhanced the uptake of lipids (Figure 4H), and the 'FASN inhibitors' caused a reduction in the intracellular lipid content (Figure 4H).

Overall, we demonstrated for the first time that vaccine MPLA FastDC from obese donors secretes high levels of IL‐10 and treatment with inhibitors of lipid metabolism targeting lipases and/or FASN reduces IL‐10 secretion removing an autocrine and paracrine inhibitory loop (Figure S6). Thus, this study suggests a strategy to improve the functionality of vaccine FastDC, particularly for the obese population that represents a large percentage of the (Western) world population and is known to have reduced responses to vaccination^2^ as well as an increased risk to develop different types of diseases including cancer.^3^

## CONFLICT OF INTEREST

The authors declare no competing financial interests.

## FUNDING INFORMATION

This work was supported from the Mildred Scheel Foundation (grant number: 70113311).

## Supporting information

Supporting information.
Figure S1. Effects of orlistat presence during maturation on MPLA FastDC phenotype and function.
After 24 h differentiation, FastDC were stimulated with the MPLA cocktail alone or together with orlistat (+ orli mat). (A) Expression of the indicated molecules on mature FastDC was determined by flow cytometry and is shown as the mean ± SE of the x‐fold increases in MFI over immature FastDC from 4 different donors. (B‐D) Autologous PBL were co‐cultured with the various MPLA FastDC for 18 h and evaluated for their ability to degranulate in response to K562 (B) and RCC53 (C) as well as to secrete IFN‐γ (D). Shown are the mean ± SE of the percentages of degranulating / IFN‐γ secreting cells among the indicated immune effector cells from four different donors after removal of spontaneous degranulation/background staining obtained by incubation of the effector cells alone. A to D: *, *p* < 0.05 in paired *t*‐test.

Supporting information.
Figure S2. Effects of orlistat on gold standard FastDC.
Monocytes were differentiated in the presence (+orli d0) or absence of orlistat and then matured with the gold standard cocktail. (A) Expression of the indicated maturation markers is shown as x‐fold increases in the MFI with respect to immature FastDC. (B‐D) PBL were stimulated for 18 h with the gold standard FastDC differentiated or not in the presence of orlistat and then evaluated for degranulation in response to K562 (B) or RCC53 (C) as well as secretion of IFN‐γ (D). Shown are the mean ± SE from six different donors. A to D: *, *p* < 0.05 in paired *t*‐test

Supporting information.
Figure S3. Effects of orlistat on FastDC cytokine secretion.
Supernatants from mature MPLA FastDC were evaluated for IL‐12p70 (A) and IL‐10 content (B). Shown are the mean ± SE of the cytokines´ concentrations from the 17 donors as well as their ratio (C). A to C: *, *p* < 0.05 in paired *t*‐test.

Supporting information.
Figure S4. Comparison of the effect of IL‐10 blockade at different time points.
Normalised frequencies of IFN‐γ secreting CD56br NK cells for each of the five donors of Figure 3 whose MPLA FastDC underwent in parallel all treatments, namely were left untreated (set to 0%), differentiated in the presence of orlistat (set to 100%, highlighted by the dashed line) or incubated with the anti‐IL‐10 blocking Ab from the start of their differentiation (+ aIL‐10 d0), during maturation (+ aIL‐10 mat) or during the co‐culture with autologous PBL (w aIL‐10 culture). Dotted lines highlight the brake of the y‐axis.

Supporting information.
Figure S5. Effects of recombinant IL‐10 during differentiation with orlistat.
Monocytes were differentiated in the presence of orlistat (orli d0) alone or together with recombinant IL‐10 (orli + recIL10 d0). Based on the data of figure 2H, 2 ng/ml recIL‐10 was added on day 0. After maturation with the MPLA cocktails, the FastDC were used to stimulate autologous PBL. After 18 h, the PBL were evaluated for secretion of IFN‐γ (A) as well as degranulation in response to K562 (B) or RCC53 (C). Shown are the mean ± SE from five different donors. A to C: *, *p* < 0.05 in paired *t*‐test.

Supporting information.
Figure S6. Effects of lipid inhibitors on FastDC.
Monocytes from obese donors tend to produce more IL‐10 upon stimulation with the MPLA cocktail. Differentiation in the presence of lipid inhibitors induces different effects including a reduced secretion of IL‐10, which results in an enhanced functional interaction with immune effector cells, in particular with NK cells, leading to enhanced IFN‐γ secretion, proliferation and cytotoxicity.
