# Supplementary figures and images for: Enhanced function of vaccine dendritic cells from obese donors upon inhibition of the lipid metabolism
Source: Clin Transl Med. 2022 Feb 25;12(2):e557. doi: 10.1002/ctm2.557 (PMC8874345; doi:10.1002/ctm2.557)

# Supplemental Figure 1

■ MPLA    ▨ + orli Mat

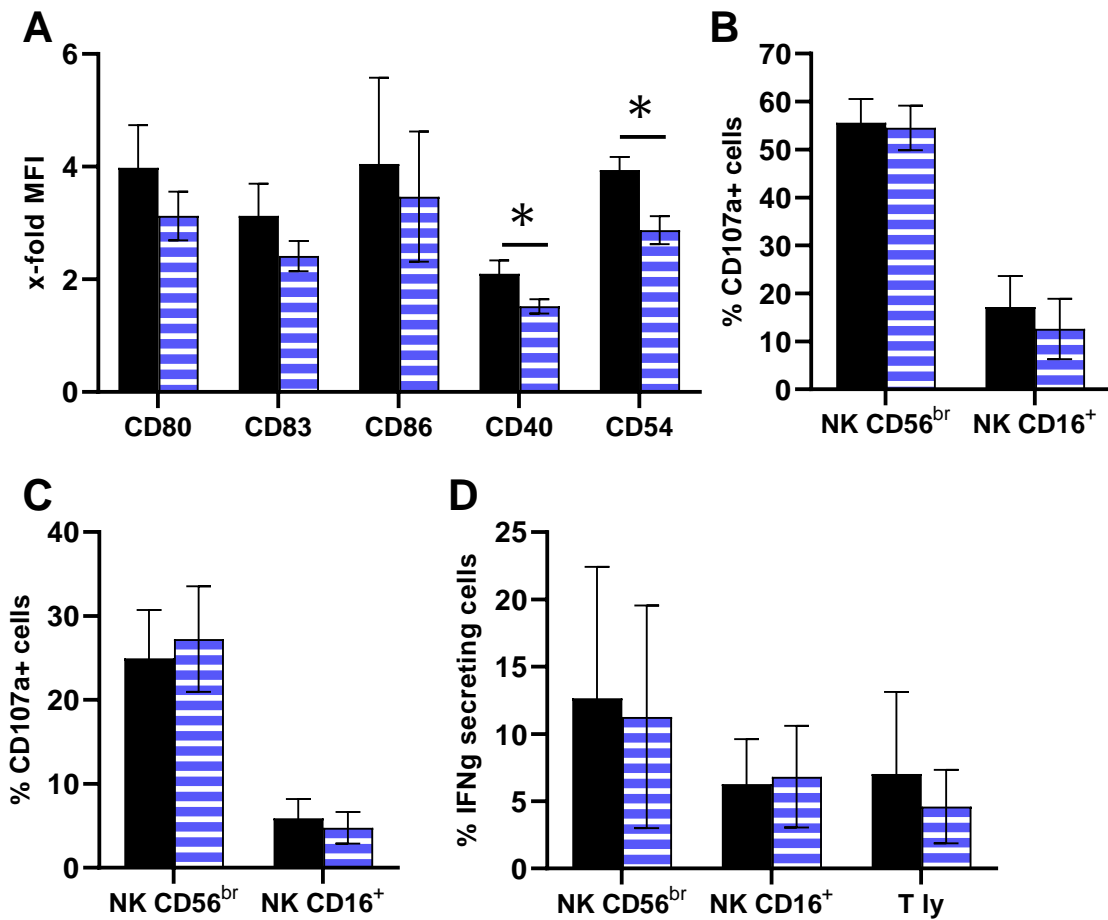

Supplement: Supplementary file 1 — Supporting information. Figure S1. Effects of orlistat presence during maturation on MPLA FastDC phenotype and function. After 24 h differentiation, FastDC were stimulated with the MPLA cocktail alone or together with orlistat (+ orli mat). (A) Expression of the indicated molecules on mature FastDC was determined by flow cytometry and is shown as the mean ± SE of the x‐fold increases in MFI over immature FastDC from 4 different donors. (B‐D) Autologous PBL were co‐cultured with the various MPLA FastDC for 18 h and evaluated for their ability to degranulate in response to K562 (B) and RCC53 (C) as well as to secrete IFN‐γ (D). Shown are the mean ± SE of the percentages of degranulating / IFN‐γ secreting cells among the indicated immune effector cells from four different donors after removal of spontaneous degranulation/background staining obtained by incubation of the effector cells alone. A to D: *, p < 0.05 in paired t‐test. [file CTM2-12-e557-s002.pdf]

# Supplemental Figure 2

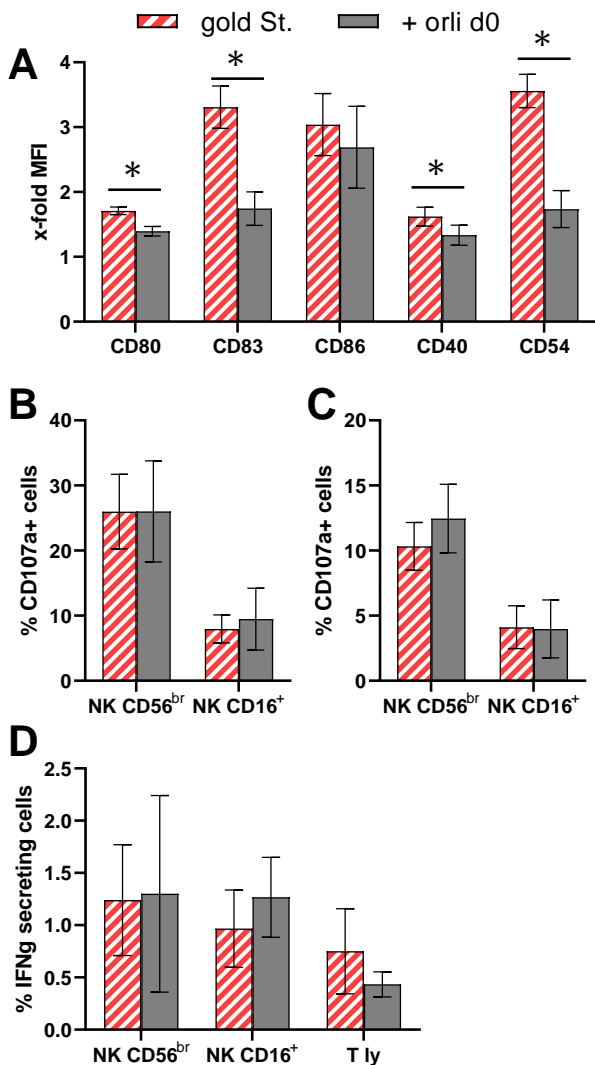

Supplement: Supplementary file 2 — Supporting information. Figure S2. Effects of orlistat on gold standard FastDC. Monocytes were differentiated in the presence (+orli d0) or absence of orlistat and then matured with the gold standard cocktail. (A) Expression of the indicated maturation markers is shown as x‐fold increases in the MFI with respect to immature FastDC. (B‐D) PBL were stimulated for 18 h with the gold standard FastDC differentiated or not in the presence of orlistat and then evaluated for degranulation in response to K562 (B) or RCC53 (C) as well as secretion of IFN‐γ (D). Shown are the mean ± SE from six different donors. A to D: *, p < 0.05 in paired t‐test [file CTM2-12-e557-s004.pdf]

# Supplemental Figure 3

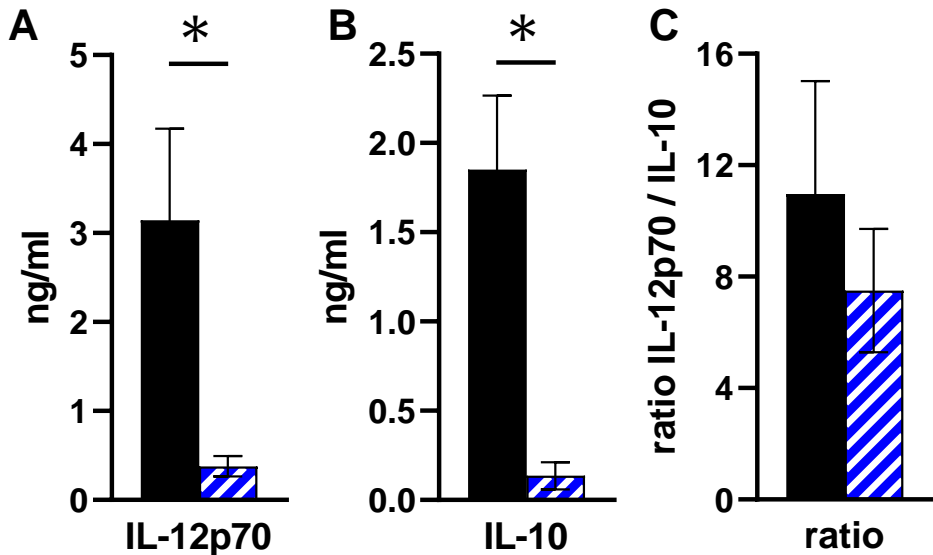

Supplement: Supplementary file 3 — Supporting information. Figure S3. Effects of orlistat on FastDC cytokine secretion. Supernatants from mature MPLA FastDC were evaluated for IL‐12p70 (A) and IL‐10 content (B). Shown are the mean ± SE of the cytokines´ concentrations from the 17 donors as well as their ratio (C). A to C: *, p < 0.05 in paired t‐test. [file CTM2-12-e557-s003.pdf]

# Supplemental Figure 4

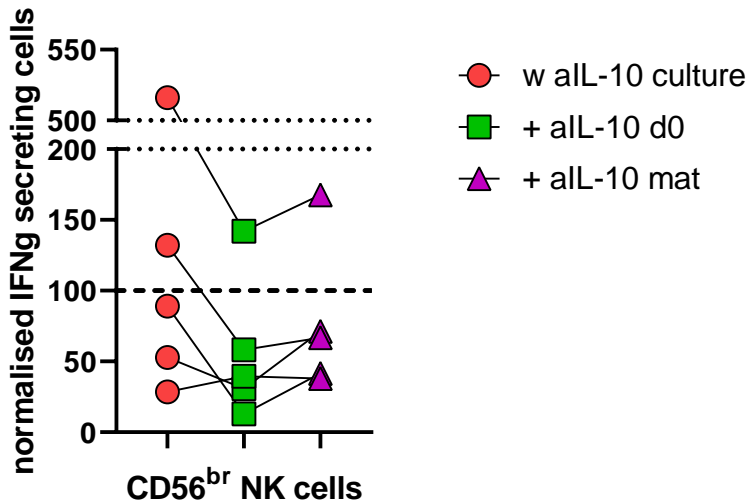

Supplement: Supplementary file 4 — Supporting information. Figure S4. Comparison of the effect of IL‐10 blockade at different time points. Normalised frequencies of IFN‐γ secreting CD56br NK cells for each of the five donors of Figure 3 whose MPLA FastDC underwent in parallel all treatments, namely were left untreated (set to 0%), differentiated in the presence of orlistat (set to 100%, highlighted by the dashed line) or incubated with the anti‐IL‐10 blocking Ab from the start of their differentiation (+ aIL‐10 d0), during maturation (+ aIL‐10 mat) or during the co‐culture with autologous PBL (w aIL‐10 culture). Dotted lines highlight the brake of the y‐axis. [file CTM2-12-e557-s001.pdf]

# Supplemental Figure 5

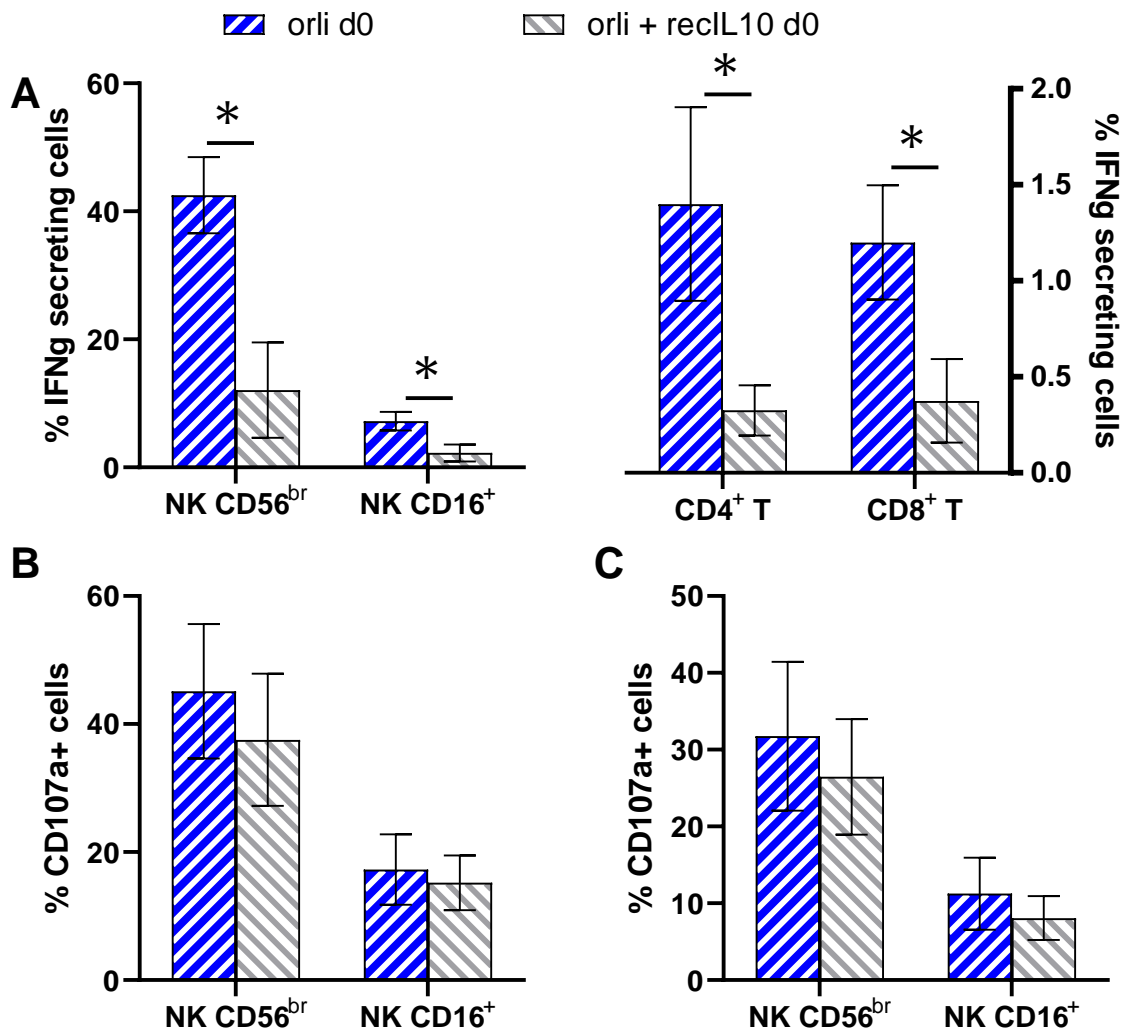

Supplement: Supplementary file 5 — Supporting information. Figure S5. Effects of recombinant IL‐10 during differentiation with orlistat. Monocytes were differentiated in the presence of orlistat (orli d0) alone or together with recombinant IL‐10 (orli + recIL10 d0). Based on the data of figure 2H, 2 ng/ml recIL‐10 was added on day 0. After maturation with the MPLA cocktails, the FastDC were used to stimulate autologous PBL. After 18 h, the PBL were evaluated for secretion of IFN‐γ (A) as well as degranulation in response to K562 (B) or RCC53 (C). Shown are the mean ± SE from five different donors. A to C: *, p < 0.05 in paired t‐test. [file CTM2-12-e557-s006.pdf]

Supplemental Figure 6

Obese MPLA FastDC

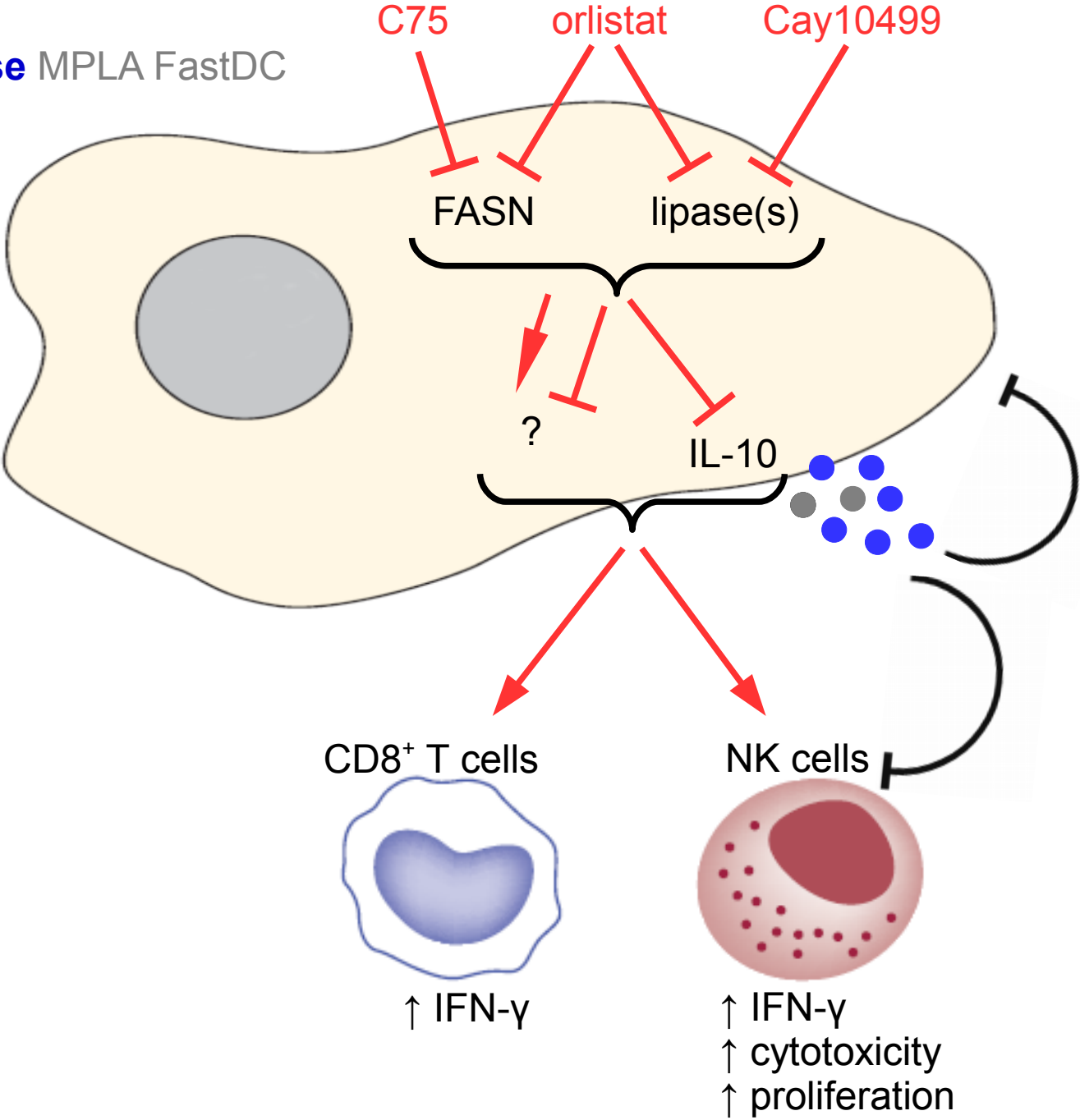

Supplement: Supplementary file 6 — Supporting information. Figure S6. Effects of lipid inhibitors on FastDC. Monocytes from obese donors tend to produce more IL‐10 upon stimulation with the MPLA cocktail. Differentiation in the presence of lipid inhibitors induces different effects including a reduced secretion of IL‐10, which results in an enhanced functional interaction with immune effector cells, in particular with NK cells, leading to enhanced IFN‐γ secretion, proliferation and cytotoxicity. [file CTM2-12-e557-s005.pdf]
